# Initial experience with a group presentation of study results to research participants

**DOI:** 10.1186/1745-6215-9-16

**Published:** 2008-03-21

**Authors:** Andrew L Avins, Stephen Bent, Amy Padula, Suzanne Staccone, Evelyn Badua, Harley Goldberg

**Affiliations:** 1 Division of Research, Northern California Kaiser Permanente, Oakland, California, USA; 2 General Internal Medicine Section, San Francisco VA Medical Center, San Francisco, California, USA; 3 Department of Medicine, University of California, San Francisco, California, USA; 4 Department of Epidemiology and Biostatistics, University of California, San Francisco, California, USA; 5 Osher Center for Integrative Medicine, Department of Medicine, University of California, San Francisco, California, USA; 6 Urology Section, San Francisco VA Medical Center, San Francisco, California, USA

## Abstract

**Background:**

Despite ethical imperatives, informing research participants about the results of the studies in which they take part is not often performed. This is due, in part, to the costs and burdens of communicating with each participant after publication of the results.

**Methods:**

Following the closeout and publication of a randomized clinical trial of *saw palmetto *for treatment of symptoms of benign prostatic hyperplasia, patients were invited back to the research center to participate in a group presentation of the study results.

**Results:**

Approximately 10% of participants attended one of two presentation sessions. Reaction to the experience of the group presentation was very positive among the attendees.

**Conclusion:**

A group presentation to research participants is an efficient method of communicating study results to those who desire to be informed and was highly valued by those who attended. Prospectively planning for such presentations and greater scheduling flexibility may result in higher attendance rates.

**Trial Registration Number:**

Clinicaltrials.gov #NCT00037154

## Background

Research participants often wish to know the results of the studies in which they have taken part [1-6]. Though data on this issue are scant, it appears that providing the results of research studies to participants is not typical [5,7,8] leaving many participants, who have born the risks and burdens of the study, without the information they desire.

It is unclear why more participants are not informed about the results of their studies. Clearly, some may choose not to know the study findings, as they may feel burdened by the implications of receiving the inferior treatment or that they may be at increased risk for future adverse outcomes [2,5,9]. For those participants who desire to learn the results, other, more practical barriers exist. Results are typically embargoed until publication in the peer-reviewed literature, so considerable time may elapse between the end of the participant-contact phase of a study and the publication of its results [10,11]; interest of participants and of study staff in re-contacting participants may wane over this interval. Perhaps more importantly, one-on-one personal contact between participants and study personnel can be very expensive and time-consuming and, therefore, may be avoided by many investigators [12].

Despite these understandable barriers, many researchers feel that participants are entitled to have the option of being informed about study results, given their contributions during the investigation; addressing this desire would be consistent with the principle of "respect for persons" that governs much of the ethical foundation of health-related research [5,12,13]. Finding ways to permit interested participants to learn the results of their studies in ways that do not place burdensome demands on investigators and staff is essential if we are to conduct research with full respect for the participants [5].

We report here our experience with a group presentation of the results of a clinical trial and ways in which this process could be adapted and improved in future studies. Others have alluded to this method [10] and it is likely that many investigators have used this technique. However, we have not found a reference to this strategy previously and our experience suggests it may serve a useful role in many research environments.

## Methods

The STEP (*Saw palmetto *Treatment for Enlarged Prostates) study was a two-arm, randomized, double-blind, placebo-controlled clinical trial of an extract of the *saw palmetto *berry for the treatment of symptoms of benign prostatic hyperplasia (BPH), funded by the National Institutes of Health [14]. The 225 participants were men at least fifty years of age with moderate-to-severe symptoms of BPH who were randomized to a *saw palmetto *extract or an identical-appearing placebo and followed for changes in symptoms and measures of urinary flow for one year. The trial showed that the herbal extract was not superior to placebo in improving symptoms or urinary function. The study was approved by the institutional review boards (IRB's) of the University of California, San Francisco, and the Kaiser Foundation Research Institute; all participants gave informed consent.

Participants were recruited from the San Francisco Veterans Affairs Medical Center (SFVAMC) and the Kaiser Permanente, Northern California (KPNC) health plan. Immediately following the termination of clinical activities, all participants were mailed a letter informing them of their randomization assignment (no study results were contained in this letter).

Following termination of the trial, the study investigators decided to hold meetings to present the results to the participants. Soon after publication of the trial findings, participants were sent a letter inviting them back to the research center for a presentation describing the results and were also offered an opportunity to receive a copy of the published paper [14] and an accompanying editorial [15]. Participants were asked to call the research center if they were interested in attending the meeting or desired to receive a copy of the manuscript. Two meetings were held, one for participants recruited from each of the two clinical sites (KPNC and SFVAMC). IRB approval for these post-trial meetings was obtained from one institution and waived by the other. The first meeting, for SFVAMC participants, was held 2 months after publication of the results (22 months after the end of the study) and the second meeting, for KPNC participants, was delayed until 9 months after publication (31 months after the end of the trial; the second meeting was delayed because of investigator availability, the need for IRB approval, and space availability). A survey was conducted at the second presentation that assessed the value and perceptions of the meeting, addressing knowledge learned about the study from the presentation and the published paper; no formal validation of the survey instrument was conducted. All responses to the survey items were measured on 7-point Likert scales.

At each presentation, copies of the published manuscript were provided to attendees. A forty-minute overview was then presented by the principal investigator. The presentation began with an overview of the disease process (BPH) and the rationale for conducting the study. This information was followed by a description of the study methods, with careful explanation of randomization, blinding, and placebos. The study results were then presented, using the tables and figures from the published manuscript [14]. Finally, the presenter provided his view of the implications of the results, an overview of future research needs, and a statement of gratitude to the participants for the knowledge they helped provide for patients with BPH. Participants were encouraged to ask questions throughout the presentation and the investigator remained after the conclusion of the formal presentation to answer individual concerns.

## Results

Attendance at the two meetings was relatively low. For the first meeting, 58 letters of invitation were mailed and 13 participants expressed interest in attending but only 3 participants actually attended the presentation. For the second meeting, 167 invitations were mailed and 30 participants expressed a desire to attend though only 17 participants were present at the meeting (several of the non-attendees cited scheduling conflicts with the meeting time). There was a great deal of energetic discussion among the participants during and after the presentations and the attendees asked numerous questions during the meetings. The questions dealt with a desire for more information about BPH, the rationales for study methods (randomization, placebos, etc.), the interpretation of the data (including the statistical aspects), and plans for future research.

All attendees of the second meeting completed the meeting questionnaire and responses to this survey revealed that virtually all participants found the presentation valuable (Table 1). Responses to items assessing reaction to the meeting were uniformly positive and all respondents thought that future studies should include this type of informational meeting. Informal comments to the presenter after the meeting indicated that several participants were very grateful for the opportunity to receive the results and interact with the investigators and other participants. Among the eight respondents who also had received a copy of the published paper prior to the meeting, the mean response to the question, "Did you find the information contained in the article understandable?" was significantly lower than the mean response to the question, "How well do you feel you've understood the results of the STEP study now [after the meeting]?" (5.3 vs. 6.7, *p *= 0.04).

**Table 1:** Survey of participants attending post-trial meeting.

**Question**	**Lower Anchor (= 1)**	**Upper Anchor (= 7)**	**Mean**	**95% C.I.**
How useful did you find this presentation?	Not useful at all	Extremely useful	6.6	6.3 – 7.0
Did this presentation provide additional information beyond what you already knew from the news media?	No	Yes	6.7	6.4 – 7.0
How well do you feel you've understood the results of the STEP study now?	Not well at all	Extremely well	6.7	6.4 – 7.0
Do you feel that you had all your questions answered about the STEP study?	No	Yes	6.8	6.5 – 7.0
Did your experience with the STEP study make you more or less interested in participating in future research studies?	Much less interested	Much more interested	6.3	5.5 – 7.0
Did your experience with this meeting make you more or less interested in participating in future research studies?	Much less interested	Much more interested	6.4	5.6 – 7.0
Should future research studies offer meetings such as this one?	No	Yes	6.9	6.8 – 7.0
If you received a copy of the published scientific article before this meeting (N = 8):				
Did you find the information contained in the article understandable?	Hard to understand	Easy to understand	5.3	4.3 – 6.2
Did you find the information contained in the article useful?	Not useful at all	Extremely useful	6.0	5.2 – 6.8

Mean responses to survey questions following meeting presentation (N = 17).

Abbreviations:

C.I. = confidence interval

STEP = Saw palmetto treatment for enlarged prostates (study)

## Discussion

Though little empiric data exist, it is likely that many research participants prefer not to learn the results of the studies in which they have taken part [2,5,9]. Others, however, may be quite interested in obtaining this information but several practical barriers have been identified that may impede providing detailed trial results to individual participants [5,7,10,12].

We found that a group presentation of study results was effective in communicating the results to some participants and was extremely well received by those who attended. Among the attendees, views of the value of the presentation were uniformly enthusiastic and virtually all felt that the meetings achieved the goals of providing the results in an easily understood format and affording an opportunity to have their post-study questions answered. It should be noted, however, that the low number of participants who attended the meeting makes generalization of our findings to other settings difficult.

Implementation of a group presentation is fairly straightforward. Most investigators will have prepared presentations for other venues (e.g., scientific meetings) that can be easily adapted to a lay audience; the grant proposal, IRB application, and published manuscripts also provide a ready source of tables and graphs for presentation. Perhaps the greatest barrier is finding available presentation space and the work of organizing the meeting itself (which may be made more difficult, as in this case, by the fact that the study has concluded and the research staff are no longer available to assist).

As noted, attendance at the meetings was relatively low. We believe there were several reasons for this, most of which can be addressed by better organizational efforts. First, the meetings were held several months after release of the study results. In our case, this was the result of conceiving the idea for the presentation relatively late, issues in obtaining presentation space, lack of administrative support for planning and conducting the presentation, and delays in obtaining IRB approval. It is possible that presentations given soon after release of findings (and, therefore, closer to the time of study closeout) might be of greater appeal to participants, whose interest in the results may wane over time. Second, we offered only one presentation opportunity for each recruitment site; providing more than one meeting might have allowed more participants to attend (as noted, several participants contacted us, expressing a desire to attend but cited scheduling conflicts as the reason for not attending the presentation). Finally, we did not envision this meeting at the beginning of the study, when we could have provided more anticipation for participants and raised expectations and awareness of this opportunity. It should be noted that we did provide each participant with his randomization assignment after study closeout. While this was appreciated by many participants (and we received no negative reactions to this notification), we may have erred in not giving participants the choice of whether to receive this information or not [1].

While our experience suggests that, with some improvements, this method is efficient in providing detailed study results and opportunity for interaction with minimal burden to study investigators, there are limitations. In particular, this technique may not be appropriate for studies of sensitive diagnoses in which anonymity may be important to participants. Even for other diagnoses, some participants may be uncomfortable appearing in a group situation with other participants, and alternatives, such as individual meetings, telephone calls, or mailing results (published papers and/or detailed summaries), may be more desirable and should also be considered. Given the increasing numbers of individuals with internet experience, one could also consider internet-based distribution of study results through a secure website and/or posting of an anonymized "frequently-asked-questions" site for more interactive distribution of study information to participants. Combinations of these methods may provide the optimal approach to addressing participants' interest in obtaining study results. It is noteworthy that, among those few respondents (n = 8) who both attended the meeting and were provided with the published paper in advance, comprehension of the study results was significantly better as a result of the presentation compared to the published manuscript, suggesting that technical articles from the medical literature may be less comprehensible to participants than information provided through in-person contact.

While we did not consider it essential to have personal contact with participants in order to deliver study results, our experience indicated that the group presentation was highly valued by virtually all of the attendees, given the results of the survey and the animated level of discussion between the participants and the investigator, and among the participants themselves. Furthermore, many participants seemed to value the queries of other participants since specific issues may not have occurred to some participants and some individuals may feel reticent to ask questions of the researchers and appreciate the candidness of other participants in questioning the investigators. Interestingly, most attendees responded that the experience of the meeting made them more interested in participating in future studies, an important ancillary benefit of promoting this type of contact with study participants.

## Conclusion

We found that a group presentation of study results was highly valued by a select group of study participants, and provided a relatively efficient means of providing results to these individuals. Several opportunities for improving our implementation are evident and, with further experience, greater attendance may result in more participants feeling that their interests and contributions were well served by researchers.

## Competing interests

The author(s) declare that they have no competing interests.

## Authors' contributions

The presentation was conceived through discussion among all authors. ALA conducted the meetings and wrote the first draft of the manuscript. All authors participated in reviewing and revising the manuscript and approved its final version.
